# Changes in phosphorus fractions associated with soil chemical properties under long-term organic and inorganic fertilization in paddy soils of southern China

**DOI:** 10.1371/journal.pone.0216881

**Published:** 2019-05-10

**Authors:** Waqas Ahmed, Huang Jing, Liu Kaillou, Muhammad Qaswar, Muhammad Numan Khan, Chen Jin, Sun Geng, Huang Qinghai, Liu Yiren, Liu Guangrong, Sun Mei, Li Chao, Li Dongchu, Sehrish Ali, Yodgar Normatov, Sajid Mehmood, Huimin Zhang

**Affiliations:** 1 National Engineering Laboratory for Improving Quality of Arable Land, Institute of Agricultural Resources and Regional Planning, Chinese Academy of Agricultural Sciences, Beijing, China; 2 National Observation Station of Qiyang Agri-ecology System, Institute of Agricultural Resources and Regional Planning, Chinese Academy of Agricultural Sciences, Qiyang, Hunan, China; 3 National Engineering and Technology Research Centre for Red Soil Improvement, Jiangxi Institute of Red Soil, Nanchang, China; 4 Soil and Fertilizer & Resources and Environmental Institute Jiangxi Academy of Agricultural Sciences, Jiangxi, China; 5 Hunan Institute of Soil and Fertilizer, Changsha, China; 6 Department of Soil Science, Gulistan state University, Gulistan City, Uzbekistan; 7 Guangdong Provincial Key Laboratory for Radionuclides, Pollution Control and Resources, School of Environmental Science and Engineering, Guangzhou University, Guangzhou, China; CSIRO, AUSTRALIA

## Abstract

The identification of phosphorus (P) fractions is essential for understanding the transformation and availability of P in paddy soils. To investigate the soil P fractions associated with soil properties under long-term fertilization, we selected three fertilization treatments, including no fertilization (CK), chemical fertilizers (NPK) and chemical fertilizers combined with manure (NPKM), from three long-term experiments located in Nanchang (NC), Jinxian (JX) and Ningxiang (NX). The results showed that chemical fertilizers combined with manure (NPKM) significantly (*P* ≤ 0.05) increased the soil total phosphorus, Olsen P and soil organic matter (SOM) by 2, 3 and 1 times, respectively, compared with the NPK treatment, and by 4, 17 and 2 times, respectively, compared with the CK treatment. NPKM significantly increased the grain yield compared with CK and NPK at all sites. The apparent P balance with NPK was higher in NC and NX but lower in JX compared with NPKM. Hedley fractionation revealed the predominance of most of the organic and inorganic phosphorus (Po and Pi) fractions with long-term fertilization, especially with the NPKM treatment, at all sites. The nonlabile P pool decreased by 14% and 18% whereas the moderately labile P pool proportions increased by 3 and 6 times with the NPK and NPKM treatments, respectively, compared to the CK treatment. The labile P pool showed a significant positive relationship with the SOM, total P and Olsen P contents. The moderately labile P was positively correlated with the total P and Olsen P. A significant positive correlation was observed between soil pH and the nonlabile P pool. Redundancy analysis revealed that the moderately labile P fraction (HCl dil. Pi fraction) was remarkably increased by the NPKM treatment and significantly correlated with the soil pH and total P concentration. The labile P fraction (NaHCO_3_-Pi) showed a strong relationship with the Olsen P and total P. However, the residual P fraction was negatively correlated with the HCl. dil. Pi fraction. We concluded that NPKM application improved P availability by many folds compared to NPK, which could lead to environmental pollution; therefore, the rate of combined application of manure and chemical fertilizer should be reduced compared to chemical fertilizer inputs to minimize the wastage of resources and environmental P losses.

## Introduction

Phosphorus (P) ranks as the second most essential nutrient after nitrogen and plays a vital role in plant growth. The availability of P in the soil depends on the P fractions, which influence the primary productivity of agricultural ecosystems [1]. In long-term fertilization, P is adsorbed and precipitated with iron, aluminum and calcium in soils, which become unavailable for plant uptake [2–4]. This results in the rapid formation of nonlabile P forms after P incorporation [5].

Most of the acidic soils in southern China are P deficient, and P fertilizer application is a common practice for overcoming the P deficiency, which causes extreme environmental problems, e.g., soil acidification, soil hardening and P leaching from the soil, ultimately reducing the soil fertility [6]. To overcome these complications, Xu [7] investigated the effects of the integrated use of organic fertilizers combined with different inorganic amendments on soil fertility and crop yield. According to their results, the combined fertilizer applications enhanced the available P concentrations and crop production. The use of inorganic fertilizers could be reduced by using organic manure to improve soil P fertility [8–10]. Moreover, these organic amendments provide both macro and micronutrients to the soil and improve its physicochemical properties [11–13].

Paddy soils undergo long-term waterlogged conditions that reduce the soil redox potential and change the P sorption and transformation [14, 15]. This low redox potential may induce changes in precipitated iron (III) phosphate to soluble iron (II) phosphate. The subsequently enhanced P available in the soil may not only assist in plant growth but also may allow P to migrate to the surrounding water pathways. Organic and inorganic phosphorus fertilization can result in varied rates of total P accumulation and create a shift in soil P fractions in the top layer of paddy soils that could provide valuable knowledge about the effects of phosphorus additions in soil phosphorus transformations [16, 17]. Therefore, exploring the status of phosphorus fractions in response to different long-term organic and inorganic fertilization in paddy soils is very important for obtaining a better understanding of P behaviors in paddy soils.

P fractions and their distributions in the soil change with the addition of different types of organic manures, such as fresh animal manure and compost [18–20]. The chemistry of soil is potentially affected by amendments with animal manure and composts, which alter both the amounts and distribution of different soil phosphorus fractions. Inorganic forms of P, such as brushite (CaHPO_4_.2H_2_O) and new berryite (MgHPO_4_.3H_2_O), are found in composted litter and categorized as moderately labile P [21].

Compared with other nutrients, P is the least available to plants and is less mobile in soil due to its adsorption to Fe and Al in paddy fields, particularly in acidic soil [2–4]. Moreover, different bio-availabilities of the geochemical fractions of soil organic and inorganic P can be changed under acidic soil conditions [22, 23]. To observe the various P fractions and P dynamics in soil, various fractionation approaches with different chemical sequential extractions can be adopted [24, 25]. However, the long-term effects of different fertilization treatments on P fractions and availability in different soils in China are less focused.

To assess the build-up of P in the soil, the fractionation methodology defined by Hedley et al. [22] is being used broadly. Fractionation schemes separate the different phosphorus fractions according to nature, either inorganic or organic and by desorption/dissolution, using different extractants with low to high desorption power and dissolution [26]. However, under subtropical conditions, a few studies have compared inorganic versus organic fertilization effects on P fractions in paddy soil, especially under long-term fertilization. Therefore, the aims of this study are (I) to determine the status of phosphorus fractions in paddy soils under long-term inorganic and manure application in southern China to obtain a better understanding of P behavior and (II) to estimate the relationship among phosphorus fractions and the influencing soil chemical properties.

## Materials and methods

### Site description

**Fig 1:**
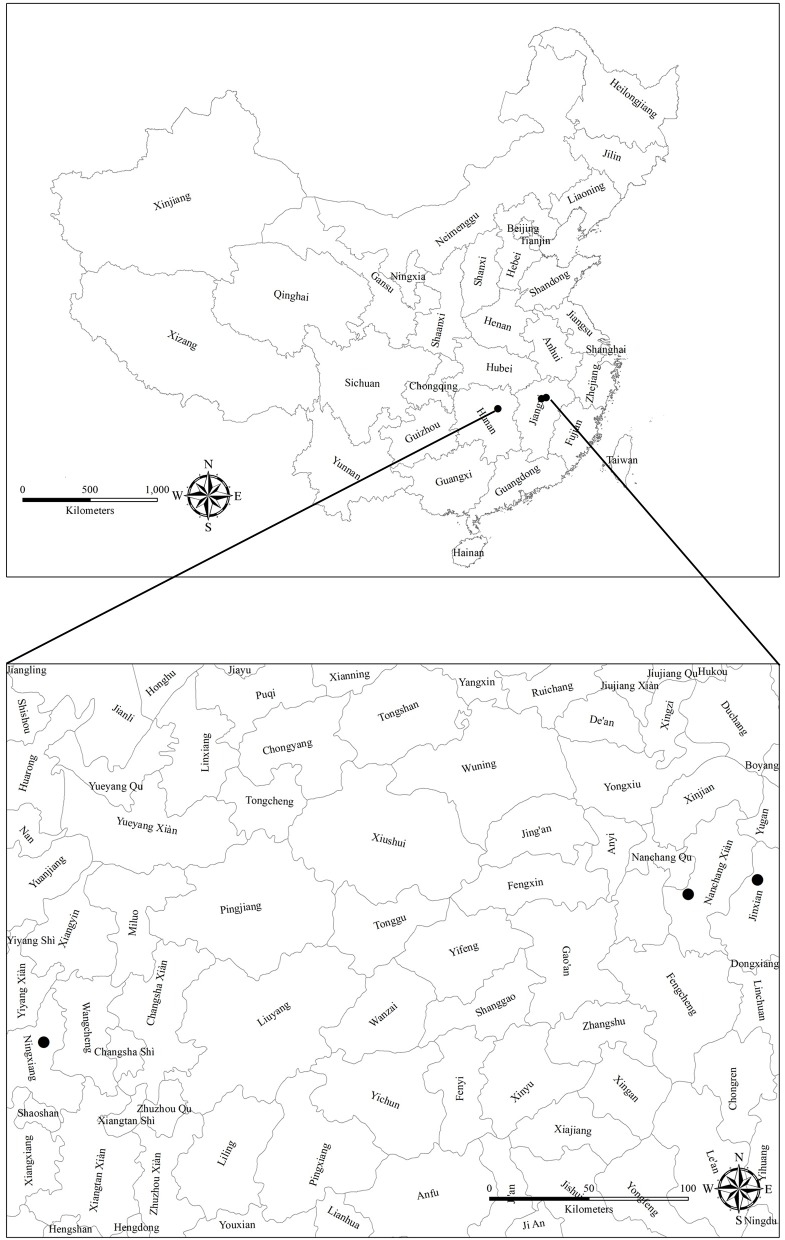
Locations of the long-term fertilization experiments in three typical croplands in China.

The three sites under long-term fertilization selected for this study are located in Nanchang (NC) which belongs to Soil, Fertilizer & Resources and Environment Institute, Jiangxi Academy of Agricultural Sciences, Jinxian (JX) and Ningxiang (NX) that belongs to Red Soil Institute of Jiangxi province, China (Fig 1). Research at these experimental sites was carried out with the consent of the Institute of Agricultural Resources and Agricultural regionalization, Chinese Academy of Agricultural Sciences, Beijing, China, under the cooperative research agreement. The basic soil physicochemical properties and climatic conditions of all the sites are given in Table 1. The NC and NX long-term experiments were located in the southern part of China, in an area with a mild subtropical climate and red soil (Ferralic Cambisol), with a heavy clayey texture, whereas the JX long-term experiment was also located in southern China but in a subtropical monsoon climate. The soil is categorized as red soil, containing 36% clay.

**Table 1:** Locations, climate conditions and initial surface soil properties of the three long-term experimental sites.

Parameters	Nanchang	Jinxian	Ningxiang
Initiation year	1984	1981	1986
Latitude (N)	28.57	28.59	28.25
Longitude (E)	115.94	116.3	112.59
Climate	MT*	SM*	SM*
Mean annual temperature (°C)	17.5	18.1	16.8
Mean annual precipitation (mm)	1600	1537	1554
Cropping system	R-R*	R-R*	R-R*
Soil classification in FAO	Eutric cambisol	Eutric cambisol	Eutric cambisol
Soil texture	Clay loam	Clay loam	Clay loam
Soil pH	6.1	6.9	6.5
SOM (g kg^-1^)	25.6	28.3	29.4
TN (g kg^-1^)	1.4	1.5	2.1
AN (mg kg^-1^)	81.6	144	143
TP (g kg^-1^)	0.5	0.5	0.6
AP (mg kg^-1^)	20.8	9.5	12.9
TK (g kg^-1^)	4.4	12.5	20.6
AK (mg kg^-1^)	35.3	81.4	33.3
Fe_d_ (g kg^-1^)	34.4	54.9	31.7
Al_d_ (g kg^-1^)	6.7	10.1	5.1

* Abbreviations: MT: monsoon temperate, SM: subtropical monsoon, R-R: rice-rice, SOM: soil organic matter, TN: total nitrogen, AN: available nitrogen, TP: total phosphorus, AP: available phosphorus, TK: total potassium, AK: available potassium, Fe_d_: Dithionite-citrate bicarbonate extractable Fe and Al_d_: Dithionite-citrate bicarbonate extractable Al.

### Cropping practices and fertilization treatments

The NC, JX and NX long-term experiments have a double-cropping system with two crops per year, i.e., rice-rice; these sites have a typical subtropical climate with two distinct growing seasons: early rice (March–July) and later rice (July–October). In our study, we analyzed only the early rice data for all three sites. The experiments consisted of different inorganic fertilization regimes and combined organic and inorganic fertilizer inputs. The treatments included no fertilization (control/CK); inorganic nitrogen, phosphorus, and potash (NPK); and manure combined with inorganic fertilizers (NPKM). The treatments were separated by cemented ridges and arranged in a randomized complete block design (RCBD) with three replicates. Inorganic fertilizers were applied as urea for nitrogen (N), calcium phosphate for phosphorus (P), and potassium chloride for potassium (K) at all the experimental sites. The fertilizer application rates over the years at all three experimental sites are listed in Table 2. In the NPKM treatment, 50% inorganic fertilizer was applied, and the remaining 50% was supplemented with organic manure based on the nitrogen contents, whereas the phosphorus and potassium rates were adjusted by inorganic fertilizers. The average nutrient contents in the organic manure at NC were N 4.5 g kg^-1^, P_2_O_5_ 2.1 g kg^-1^, and K_2_O 6.2 g kg^-1^; at JX, N 6.3 g kg^-1^, P_2_O_5_ 4.5 g kg^-1^, and K_2_O 5.1 g kg^-1^; and at NX, N 17.5 g kg^-1^, P_2_O_5_ 8.3 g kg^-1^ and K_2_O 11.2 g kg^-1^. The manure application rate at NC, JX and NX were 16700 kg ha^-1^; 15000 kg ha^-1^; and 4050 kg ha^-1^. All the phosphorus fertilizers and manure were applied as a basal dose before seedling transplantation, and 50% of N and K was applied as basal application; 25%, top-dressed at the tillering stage; and another 25%, top-dressed at the panicle stage.

**Table 2:** Fertilizers input rates (kg ha^−1^) in the three long-term experiments in three typical croplands in China.

Sites^a^	Fertilizer application (N-P-K)		
	CK^b^	NPK^c^	NPKM^d^
NC	0-0-0	150–26.18–124.5	150–26.18–124.5
JX	0-0-0	90–19.56–62.25	180–39.28–124.5
NX	0-0-0	143–23.56–52.29	143–23.57–52.29

aSites: NC: Nanchang, JX: Jinxian, NX: Ningxiang.

bCK: unfertilized control.

cNPK: inorganic nitrogen, phosphorus and potassium.

dNPKM: inorganic NPK plus manure

### Soil sampling and analyses

Soil samples were taken from 0–20 cm soil depth from all the three sites after the crop harvest in July 2017 with an auger (5 cm diameter). Each sample was a composite of three random cores from each plot. The fresh samples of soil were mixed altogether, air-dried, and then sieved through a 1.0 mm sieve, after which they were placed in fixed plastic bags for analysis. The representative subsamples were ground to 0.25 mm to evaluate the organic C [27], total P [28], Olsen P (available P) [29] and P fractions [22]. The soil pH was assessed in a 1:2.5 soil: water suspension [30].

### P fractionation

The chemical species of P in the soil were analyzed by the Hedley fractionation method as modified by Tiessen and Moir [23]. Briefly, triplicate subsamples of each soil (1 g) were sequentially extracted as described: 1 g of soil was collected and shaken with 0.5 M NaHCO_3_ at pH 8.5 for 16 consecutive hours (NaHCO_3_-P), 0.1 M NaOH for 16 hours (NaOH-P), with 1 M HCl for 16 hours (diluted HCl-P), heated with 10 ml of concentrated HCl in a water bath for 10 minutes at 80°C containing 5 ml of 12 M HCl, and then conveyed to a final volume of 50 ml with distilled water (concentrated HCl-P). Finally, by using concentrated H_2_SO_4_ (300 μl per 30 mg of soil buildup subsample) at 350°C for 3 hours (rate of 4°C/min; residual phosphorus), the soil residue samples were digested. Between two continuous phases, the tubes were placed for centrifugation for 10 minutes at 25,000×g and 4°C. By using 0.45 μm cellulose nitrate filters, the supernatant was discarded, and the filters were cleaned with the extractant utilized in the accompanying step to trap additional soil particles. Both the organic and inorganic phosphorus (by using colorimetry, the difference between the total phosphorus and inorganic phosphorus contents were evaluated after persulfate digestion) levels were assessed by applying solutions of 0.5 M NaHCO_3_, 0.1 M NaOH and 12 M HCl separately.

### Statistical analysis

All statistical analyses were conducted using SPSS 20.0. Analysis of variance was used, and significant differences were evaluated using the least significant differences (LSD) test at *P* ≤ 0.05. Regression analysis was used to determine the changes in the various phosphorus pools caused by the different soil chemical properties. Redundancy analysis (RDA), a type of linear-constrained ordination based on principal component analysis, was conducted to develop the relationship between the changes in soil phosphorus fractions and chemical properties of the soil using Canoco version 5.

## Results

### Soil organic matter and pH

In the present study, the soil organic matter and pH were significantly (*P* ≤ 0.05) influenced by the site × fertilization interaction (Table 3), and the SOM was significantly increased compared to the initial soil properties before the start of the long-term experiments (Table 1). A non-significant decrease in soil pH compared with initial soil pH was observed at all the three long-term experiments, the NPKM treatment showed significant (*P* ≤ 0.05) results compared to the inorganic fertilizer (NPK) and control (CK) treatments at all sites. The SOM content was highest at NC (48.4 g kg^−1^), followed by JX (37.5 g kg^−1^) and NX (37.3 g kg^−1^). Compared with the CK treatment, the NPKM treatment increased the SOM contents by 48.4%, 37.5% and 37.3% at NC, JX and NX, respectively, whereas the NPK treatment increased the SOM contents by 41.1%, 32.5% and 24.7% in NC, JX and NX, respectively. The average SOM contents at all three sites were 24.3, 32.8 and 41.1 g kg^−1^ in the CK, NPK and NPKM treatments, respectively. The average increase in SOM content with the NPK and NPKM treatments was 34.7% and 68.9%, respectively, compared with the CK treatment among all three sites. The average soil pH was 5.8, 5.7 and 6.1 in the CK, NPK and NPKM treatments, respectively (Table 3), and the highest pH was observed in NX (6.7), followed by JX (5.9) and NC (5.7).

**Table 3:** Effect of long-term fertilization on P balance, grain yield, soil pH, SOM, total P and Olsen P concentrations.

Sites^a^	Treatments^b^	pH	SOM	Total P	Olsen P	P balance	Grain yield
		(H_2_O)	(g kg^-1^)	(g kg^-1^)	(mg kg^-1^)	(kg ha^-1^)	(kg ha^-1^)
NC	CK	5.65 ± 0.10 Ab	29.27 ± 0.20 Ca	0.36 ± 0.04 Cb	7.28 ± 0.24 Ca	-7.7 ± 1.02 Cb	1844 ± 143.2 Ca
	NPK	5.20 ± 0.08 Bb	41.08 ± 0.06 Ba	0.54 ± 0.03 Bb	61.13 ± 1.40 Ba	6.8 ± 0.98 Ab	4129 ± 144.7 Ba
	NPKM	5.71 ± 0.13 Ab	48.44 ± 1.33 Aa	1.63 ± 0.06 Ac	81.50 ± 2.18 Ab	-1.9 ± 3.19 Bc	4615 ± 181.2 Aa
JX	CK	5.59 ± 0.14 ABb	22.85 ± 0.52 Cb	0.49 ± 0.01 Ca	6.52 ± 0.53 Ca	-4.8 ± 0.45 Ca	1608 ± 352.4 Ca
	NPK	5.45 ± 0.08 Bb	32.54 ± 0.99 Bb	0.93 ± 0.02 Ba	30.07 ± 1.39 Bb	9.9 ± 0.81 Ba	2510 ± 105.8 Bc
	NPKM	5.91 ± 0.14 Ab	37.53 ± 1.59 Ab	1.63 ± 0.13 Ab	64.69 ± 1.64 Ac	21.3 ± 1.02 Aa	4533 ± 108.1 Aa
NX	CK	6.30 ± 0.05 Ba	20.89 ± 0.65 Cc	0.45 ± 0.07 Ca	4.53 ± 0.33 Cb	-5.4 ± 0.11 Ca	1631 ± 35.5 Ca
	NPK	6.54 ± 0.15 Aa	24.74 ± 1.39 Bc	0.96 ± 0.10 Ba	14.97 ± 0.16 Bc	8.5 ± 0.93 Aab	3337 ± 79.6 Bb
	NPKM	6.73 ± 0.04 Aa	37.32 ± 1.43 Ab	2.21 ± 0.15 Aa	90.80 ± 2.86 Aa	2.2 ± 0.85 Bb	4047 ± 76.1 Ab
	ANOVA						
	Soil type	***	***	***	***	***	***
	Fertilization	***	***	***	***	***	***
	S*F	***	***	***	***	***	***

a Sites: NC: Nanchang, JX: Jinxian, NX: Ningxiang

b Treatments: CK: unfertilized control; NPK: inorganic nitrogen, phosphorus and potassium; NPKM: inorganic NPK plus manure

Data followed different uppercase letters denote significant differences (*P* ≤ 0.05) between fertilization treatments at the same site (A, B, C) and lowercase letters between sites for the same fertilization treatment (a, b, c).

Significance levels

*** represents *P*<0.01

### Changes in soil P fertility

The soil total and Olsen P concentrations varied among the different treatments, and a significant increase was observed compared with initial soil properties at all the experimental sites (Table 1). The highest total P concentration was 2.2 g kg^−1^ with the application of the NPKM treatment in NX, followed by JX and NC (Table 3). Compared to the CK treatment, the NPK treatment increased the soil total P concentrations by 49.9%, 89.1% and 111%, respectively, in NC, JX and NX. The NPKM treatment increased the total P by 5, 3 and 5 times more than the NPK treatment in NC, JX and NX, respectively. The total P concentration averaged 0.4, 0.8 and 1.8 g kg^−1^ in the CK, NPK and NPKM treatments, respectively, at all three sites. Moreover, the NPKM treatment resulted in a significant (*P* ≤ 0.05) increase in the Olsen P concentration compared to the NPK and CK treatments (Table 3). The Olsen P concentration was highest in NX (90.8 mg kg^−1^), followed by NC and JX. The NPK treatment increased the Olsen P levels by 8, 5 and 3 times, whereas the NPKM treatment increased by 11, 10 and 20 times more than the CK treatment at NC, JX and NX, respectively. The average Olsen P concentration was 6.1, 35.4 and 104 mg kg^−1^ in the CK, NPK and NPKM treatments, respectively, at all three sites.

#### Soil P balance and the grain yield

The soil apparent P balance was significantly affected by the long-term fertilization. Apparent P balance in NPK was significantly greater than NPKM and CK in NX (8.5 kg ha^−1^) and NC (6.8 kg ha^−1^), except JX where NPKM (9.9 kg ha^−1^) had the highest P balance compared with NPK and CK (Table 3). Grain yield was also significantly increased by the long-term application of NPKM compared with NPK and CK at all three sites. The highest grain yield was 4615.2 kg ha ^−1^ with the application of the NPKM treatment in NC, followed by JX (4533.4 kg ha ^−1^) and NX (4047.7 kg ha ^−1^) (Table 3).

### Soil P fractions

#### P fractions affected by long-term inorganic and organic fertilizer applications

The long-term organic and inorganic fertilization management practices had various effects on the sizes of the individual phosphorus pools (Table 4). In the current study, the NPKM treatment significantly (*P* ≤ 0.05) increased the size of all the phosphorus pools except the nonlabile P fractions when compared with CK and NPK, among all three sites. The sum of all organic and inorganic P fractions depicted the same increasing trends, i.e., NPKM>NPK>CK, compared with the actual soil total P content at all three sites. The NPKM treatment showed significant (*P* ≤ 0.05) differences for all the P fractions compared to the CK and the NPK treatments. Compared to the CK and NPK treatments, the NPKM treatment showed a significant (*P* ≤ 0.05) increase in the inorganic and organic P fractions (NaHCO_3_-P, NaOH-P, dil. HCl-P and conc. HCl-P), except for the residual P contents, at all three sites. The fractionated total phosphorus (sum of all the inorganic and organic phosphorus fractions) contents among the inorganic and organic fertilization treatments for each site were reduced in the order of NPKM *>*NPK *>*CK at NC, JX and NX, respectively. NC exhibited the highest concentration of concentrated HCl-Pi and residual Pi, followed by NX and JX. JX exhibited the highest concentration of NaHCO_3_-Pi (206 mg kg^-1^), followed by NC (193 mg kg^-1^) and NX (151 mg kg^-1^). Most of the organic and inorganic fractions were found at the highest concentration at NX, i.e., NaHCO_3_-Po (51.6 mg kg^-1^), NaOH-Pi (253 mg kg^-1^), NaOH-Po (164 mg kg^-1^), HCl dil. Pi (334 mg kg^-1^), and HCl conc. Po (33.2 mg kg^-1^), followed by JX and NC (Table 4). The proportions of the abovementioned fractions under the NPK and NPKM treatments at NX were increased by 5 and 21, 4 and 19, 2 and 7, 3 and 12, and 3 and 12 times, respectively, compared to the CK treatment.

**Table 4:** The concentration of different P fractions in each fertilization treatment and site (mg kg^-1^). ANOVA significance levels of the effects of site, fertilization treatment, and the interactions between site and fertilization.

Sites	Treatments^a^	Labile P (mg kg^-1^)		Moderately labile P (mg kg^-1^)			Nonlabile P (mg kg^-1^)			
		NaHCO_3_-Pi	NaHCO_3_-Po	NaoH-Pi	NaoH-Po	Hcl. dil-P	Hcl. Conc-Pi	Hcl.Conc-Po	Res-P	Total-P
NC	CK	8.8 ± 0.31 Cb	12 ± 1.57 Ca	48.8 ± 2.01 Ca	19.6 ± 0.51 Cb	4.1 ± 0.51 Bc	4 ± 1.60 Ca	1.1 ± 0.04 Bb	151 ±1.16 Aa	249
	NPK	153 ± 1.20 Ba	30.7 ± 4.90 Ba	212 ± 0.60 Ba	27.5 ± 1.32 Bb	4.2 ± 0.27 Bc	109 ± 0.31 Ba	5 ± 0.15 Ab	97.4 ± 1.65 Ba	638
	NPKM	193 ± 1.77 Ab	45.9 ± 1.62 Ab	225 ± 2.45Ac	53 ± 1.06 Ab	13 ± 2.46 Ac	121 ± 0.68 Aa	5.1 ± 0.20 Ab	35.1 ± 0.96 Ca	692
JX	CK	30.8 ± 0.15 Ca	10.6 ± 0.23 Ca	46.9 ± 1.34 Ca	12 ± 2.05 Cc	67.4 ± 0.23 Ca	4.9 ± 0.11 Ba	1.1 ± 0.04 Cb	133 ± 0.13 Ab	306
	NPK	153 ± 1.20 Ba	31 ± 5.12 Ba	212 ± 0.60 Ba	42 ± 2.25 Ba	109 ± 0.31 Ba	5.6 ± 0.40 Bb	1.5 ± 0.61 Bc	32.7 ± 0.10 Bb	586
	NPKM	206 ± 0.46 Aa	41.6 ± 1.19Ac	236 ± 0.60 Ab	53 ± 1.73Ab	121 ± 0.68 Ab	7.3 ± 0.70 Ac	3.2 ± 0.82 Ab	12.7 ± 0.76 Cb	681
NX	CK	5.8 ± 0.46 Cc	2.4 ± 0.46 Cb	13.1 ± 0.30 Cb	23.8 ± 0.35 Ca	28 ± 0.46 Cb	0.2 ± 0.04 Cb	2.8 ± 0.52 Ca	29.4 ± 0.96 Ac	105
	NPK	18.1 ± 0.35 Bb	13.1 ± 1.65 Bb	57.8 ± 0.50 Bb	42.1 ± 0.86 Ba	90 ± 2.08 Bb	3.9 ± 0.02 Bc	8.1 ± 1.48 Ba	17.2 ± 2.55 Bc	251
	NPKM	151 ± 3.28 Ac	51.6 ± 2.08 Aa	253 ± 1.99 Aa	164 ± 1.35 Aa	334 ± 3.01 Aa	38.7 ± 0.57 Ab	33.2 ± 2.11 Aa	11.5 ± 0.93 Cb	1036
	ANOVA									
	Soil type	***	***	***	***	***	***	***	***	***
	Fertilization	***	***	***	***	***	***	***	***	***
	S*F	***	***	***	***	***	***	***	***	***

a Treatments: CK: unfertilized control; NPK: inorganic nitrogen, phosphorus and potassium; and NPKM: NPK plus manure.

Data (means ± SD, n = 3) followed by different uppercase letters denote significant differences (*P* ≤ 0.05) between fertilization treatments at the same site (A, B, and C), and lowercase letters denote significant differences (*P* ≤ 0.05) between sites for the same fertilization treatment (a, b, and c).

Significance levels

*** represents *P*<0.01

Different sequential phosphorus fractions were categorized into three pools: (1) labile phosphorus (NaHCO_3_-Pi+NaHCO_3_-Po), (2) moderately labile phosphorus (NaOH-Pi +NaOH-Po+ Dil. HCl-Pi) and (3) nonlabile or stable phosphorus (HCl conc.-Pi+ HCl conc.-Po+ residual P). The labile and moderately labile phosphorus pools in NPKM at NC were 12 and 4 times; at JX, were 6 and 3 times; and at NX, were 25 and 12 times greater than CK, respectively. In all three sites, compared to the CK treatment, the labile proportion was 10 times higher, and the sum of nonlabile P fractions was 11 times lower in the NPKM treatment (Table 4). In contrast, the nonlabile P also increased with the NPKM treatment at NC by 4% and NX by 157%. Among all three sites, the labile phosphorus, moderately labile phosphorus and nonlabile phosphorus averaged 23.4, 87.8 and 109 mg kg^−1^ in the CK treatment, respectively, and 132.6 mg kg^−1^, 265.4 mg kg^−1^ and 93.5 mg kg^−1^ in the NPK treatment, respectively. However, due to the increase of 230 mg kg^−1^ in the labile P pool and 484 mg kg^−1^ in the moderately labile P pool, the non-labile P pool decreased by 18% with the NPKM treatment compared with the CK and NPK treatments (Table 4). The labile pool in the NPK and NPKM treatments increased by 6 and 10 times and in the moderately labile P pool by 3 and 6 times, respectively, whereas the nonlabile-P pool decreased by 14% and 18% compared to the CK treatment at all sites, respectively.

#### Relationship of P fractions with other soil properties

**Fig 2:**
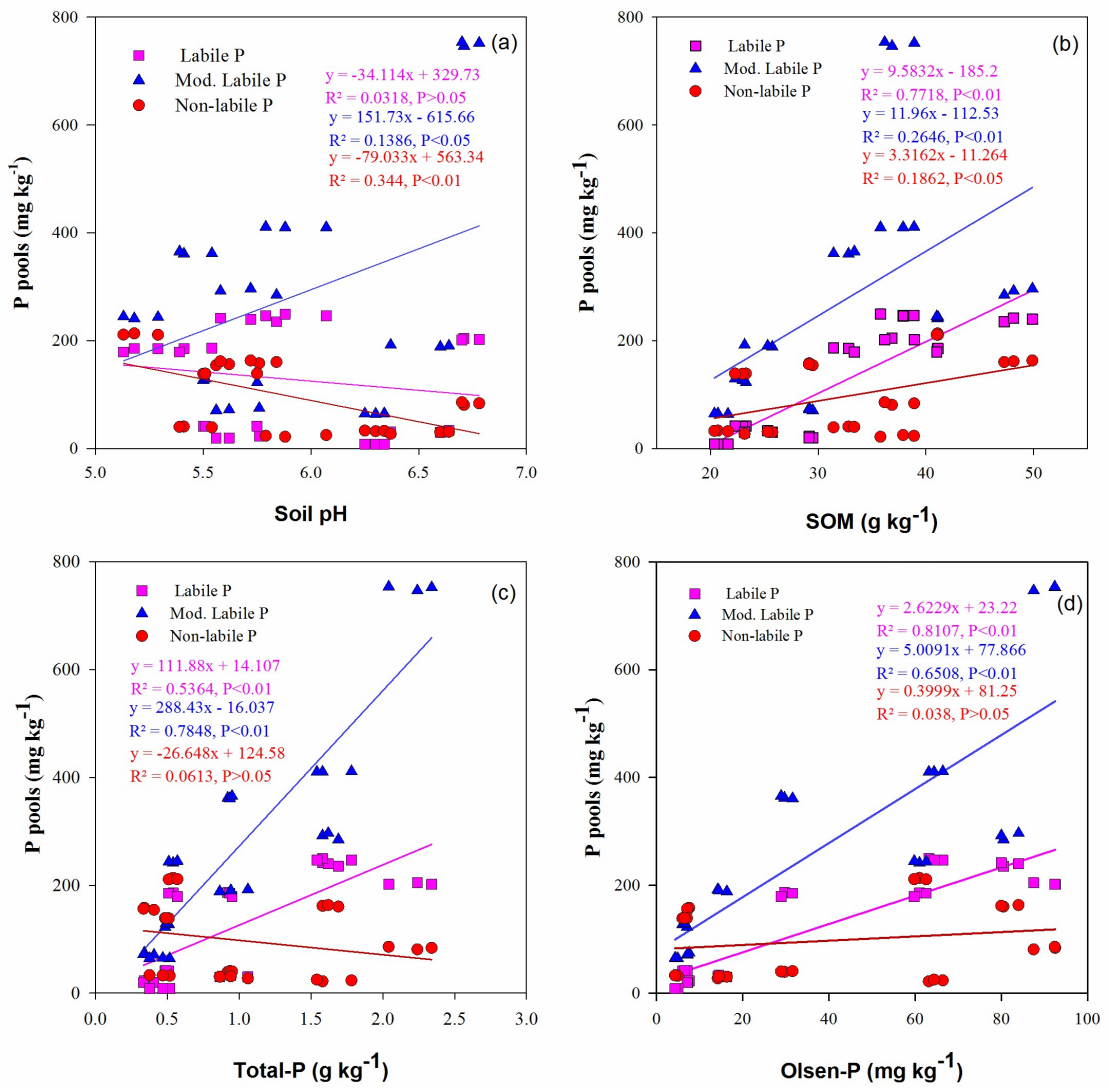
Relationship between soil properties, labile P, moderately labile P and nonlabile P fractions under long-term fertilization in paddy soils.

The relationships among the soil properties, labile P, moderately labile P and nonlabile P pools are shown in Fig 2. In the regression equation, x specifies the soil chemical property, and y specifies the changes in the soil P pool (ΔP pools) concentrations. Therefore, the regression equation slope indicates the change in the phosphorus pool (mg kg^-1^) concentration per unit of increase/decrease in the respective soil properties. The labile P pool showed a significant positive relationship with the soil organic matter content (R^2^ = 0.77, *P* ≤ 0.01), total P concentration (R^2^ = 0.54, *P* ≤ 0.01) and Olsen P levels (R^2^ = 0.81, *P* ≤ 0.01) in the soils, whereas it showed a non-significant relationship with the soil pH. The moderately labile P was positively correlated with the total (R^2^ = 0.78, *P* ≤ 0.01) and Olsen P (R^2^ = 0.65, *P* ≤ 0.01), and a significant (*P* ≤ 0.05) relationship was observed with soil pH and SOM content. The soil pH depicted a significant (*P* ≤ 0.01) negative relationship with the nonlabile P pool, whereas this pool also had a nonsignificant correlation with the soil total and Olsen P levels in the soil.

### Redundancy analysis (RDA) of the soil properties and different P fractions

**Fig 3:**
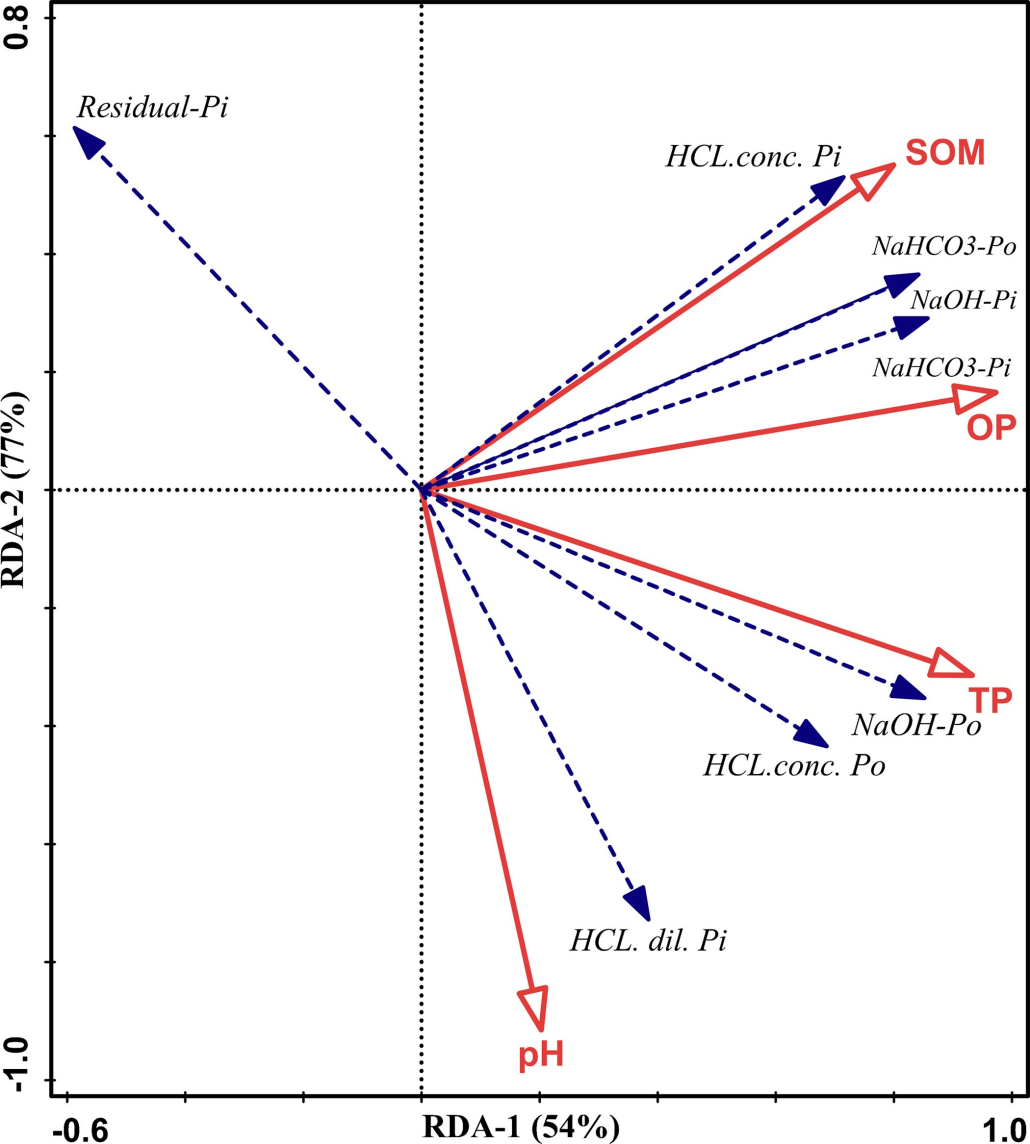
RDA analysis showing the relationship of the soil properties (SOM, OP, TP and pH) with the P fractions under long-term fertilizer management. * Abbreviations: SOM: soil organic matter, TP: total-P, and OP: Olsen P.

The relationship among the different soil phosphorus fractions and the soil chemical properties was analyzed by the RDA plot (Fig 3). The soil inorganic and organic P fractions were considered explanatory variables, and the different soil properties were considered response variables. In the RDA ordination plot, the angles between the explanatory and response variables or between the response variables themselves show their correlations, and the relationship between the centroid of a qualitative response variable and explanatory variable is also observed by projecting the centroid at a right angle to the variable. A high correlation between variables is represented by smaller angles between arrows, and positive or negative correlations are represented by the direction of the arrows. The first (RDA 1) and second (RDA 2) ordination axes accounted for 54% and 77% of the total variation between the soil phosphorus fractions and the chemical properties, respectively. The moderately labile P fraction (HCl dil. Pi) was highly influenced by the combined application of organic and inorganic fertilizers (NPKM) and was significantly correlated with the soil pH (*P* ≤ 0.01) and total P concentration (*P* ≤ 0.01). The labile P fractions (NaHCO_3_-Pi and NaHCO_3_-Po) showed a strong relationship with the Olsen P concentration (*P* ≤ 0.01) and SOM contents (*P* ≤ 0.01); however, the residual P fraction showed an opposite trend, decreasing with the application of organic and inorganic fertilizers and showing a negative correlation with soil pH, total P and the HCl dil.-Pi fraction. The SOM content exhibited a strongly positive correlation with both the labile phosphorus and moderately labile inorganic phosphorus fractions (NaHCO_3_-Pi and NaOH-Pi).

## Discussion

### Soil P fractions and soil properties

In our study, different sites and long-term fertilization significantly (*P* ≤ 0.05) influenced the organic and inorganic P fractions (Table 4), which could be the effect of different P fertilization treatments, their application rates (Table 2) and different soil properties (Table 3). The combined application of chemical fertilizers and manure showed significant (*P* ≤ 0.05) differences in the total and Olsen P concentrations compared to their initial concentrations. This might be due to the long-term addition of manure to soil, which increased the soil total phosphorus and available phosphorus contents (Table 3). Previous studies have reported that long-term chemical fertilization and manure addition increases the soil total phosphorus and available P stocks [31]. In this study, the total and Olsen P contents were highest in the NPKM treatment compared with the CK and NPK treatments. It was stated by Zhang et al [32] that an Olsen P concentration in soil ranging from 5 to 10 mg kg^−1^ would be sufficient for crop growth, whereas a concentration less than 5 mg kg^−1^ would be P-deficient, thereby affecting plant growth. In another study, Bravo, Torrent [33] reported that for optimal crop growth, the concentration of Olsen P should be above a critical level of 6 or 7 mg kg^−1^. In our present study, the NPKM treatment showed a significant (*P* ≤ 0.05) increase in the total P and Olsen P contents compared to the NPK and control (CK) treatments, showing that the combined application of inorganic (NPK) and manure fertilizer caused synergistic effects on the soil Olsen P concentrations. Similar findings were also mentioned by Garg and Bahl [34], who determined that, throughout the incubation period, organic fertilizers combined with inorganic fertilizer inputs significantly (*P* ≤ 0.05) increased the Olsen P concentrations. The long-term effects of the combined application could be due to the following reasons: (1) the applied organic manure has an additional supply of P, (2) the applied organic and inorganic P from different sources is prevented from adsorption and the formation of insoluble complexes in the soils [35, 36], and (3) the native soil phosphorus was mobilized by the organic amendments [8]. Several studies have shown that phosphorus fractions in the soil are mainly affected by the chemical properties of the soil (SOM, total P and Olsen P), physical properties (particle size distribution and soil moisture content), various microbial activities in the soils, and different agricultural management practices, especially phosphorus fertilizer applied to soils [37, 38].

In our results, the apparent P balance in NPK was significantly greater than the NPKM in NC and NX, but not in JX (Table 3). In JX, the P fertilizer application rate under NPKM was higher than that of NPK, with the P balance increasing with NPKM. In NC and NX, lower P balance was due to high P uptake under combined application of organic and inorganic fertilizers. These results are consistent with the previously reported studies [39]. Different studies documented that excessive manure application lead to the P accumulation in soil and may cause significant environmental pollution; therefore, wise management of P fertilizer application rate is essential for sustainable crop production under paddy soil conditions to minimize environmental impact [40–43]. Long-term application of NPKM significantly increased the grain yield compared with CK and NPK (Table 3). This could be because of the long-term manure addition Which overcame macronutrient deficiencies and provided beneficial effects that enhanced the availability of soil micronutrients [44]. Also, the manure addition provided beneficial conditions and nutritive substrate that are needed by plants during the grain filling period [45].

### Fractional distribution of soil P

Continuous P fertilization combined with manure significantly (*P* ≤ 0.05) increased the P accumulation. This finding is in accordance with previous studies [46, 47]. Long-term P fertilization increased all forms of Pi, including the labile Pi, moderately labile Pi, and nonlabile Pi fractions. Our results demonstrated that the long-term addition of manure to soil has a direct impact on the phosphorus status of paddy soils. Compared with the CK and NPK treatments, the NPKM treatment significantly (*P* ≤ 0.05) increased the total P accumulation in the paddy soils (Table 3). This might be due to different P fertilizers (organic and inorganic) having different effects on P accumulation and its mobility in the upper (20 cm) layer of paddy soils [47, 48].

NaHCO_3_-Pi, considered as inorganic labile P, is a biologically available form of phosphorus [49], whereas NaHCO_3_-Po is considered an easily mineralizable organic phosphorus [50]. In the current study, the two labile P fractions in the NPK and NPKM treatments accounted for 29% and 35%, 31% and 36%, and 12% and 20% of the total P pool at NC, JX and NX, respectively, as shown in Table 4, and the highest labile P fractions were in the NPKM treatment compared with the NPK and CK treatments at all sites This showed that the supply of available P capacity was better sustained by the inorganic fertilizer application combined with organic manure than that of inorganic fertilization alone. [12] stated that P precipitation with calcium phosphate decreases the P availability for plant uptake that could be mobilized by the addition of manure to the soil [8]. During the decomposition of manure, organic acids are produced, which results in the formation of P complexes along with iron and aluminum, reducing the P availability in soil. Our results are consistent with previous studies [13, 51], which found that the long-term cultivation of crops with no fertilizer application decreased the labile Pi and Po contents in the soil, and chemical fertilizer application with pig manure significantly increased the labile Pi and Po contents in the soil. In our study, the relationship between inorganic P fractions and SOM contents also showed that the addition of manure decreased the precipitation of low soluble phosphate. The average concentrations of NaHCO_3_-Pi and NaHCO_3_-Po were highest in the NC site, possibly due to the higher SOM contents in the soil, which could affect the P sorption sites as follows: (I) by creating a mask over the aluminum and iron sorption sites to stop the added phosphorus from adsorption and (II) by changing the mineral surface charges, which in return reduces the sorption sites ultimately increasing the soil P [49].

The fractions of organic (NaOH-Po), inorganic (NaOH-Pi) and HCl dil.-P were considered as the moderately labile phosphorus pool, which accounted for 38% and 42%, 60% and 62%, and 72% and 76% of the total P pool at NC, JX and NX, respectively, with the NPK and NPKM treatments, and it was the largest P pool among all sites and for all treatments. Similar to the labile P fraction, the NaOH extractable P fraction also increased with the organic treatment, NPKM, followed by the NPK and CK treatments (Table 4). Previous studies also have shown that the application of different types of organic fertilizers significantly increased the NaOH-Po pools, and this increase was due to the addition of organic fertilizer compared with inorganic fertilizers [9, 52, 53]. Our findings are also consistent with the previous results by Gichangi et al [4] and Malik et al [5], who confirmed that organic phosphorus sources predominantly possessing higher phosphorus concentrations can have stimulating effects for increasing the organic P contents in soils, and the retention of organic phosphorus may be due to the adsorption of P in organic materials, which are likely to be changed into inorganic P by plant root activities. The increase in the organic P pool in the NPKM treatment compared with the NPK and CK treatments might be due to the slow release of phosphorus from organic materials that can play a pivotal role in the cycling of soil phosphorus.

Concentrated HCl-P and residual phosphorus fractions, which are mainly composed of insoluble and stable forms of phosphorus, such as Ca-, Fe- and Al-bounded P [50, 54], represent the unavailable forms of P pools in the soil. Our study showed that the nonlabile P pool concentrations followed the opposite trend (Table 4) compared with the labile P pool. These results agree with the findings of Dobermann et al [55], who stated that P fertilizer application had little effect on the organic and residual phosphorus fractions but mainly increased the soluble inorganic P. While the residual P and HCl-Pi fractions were supposed to be sparingly available pools of phosphorus [53], the change in the concentrations of the nonlabile phosphorus fractions observed in this study suggests that these fractions might be involved in long-term phosphorus cycling. This process may result from the occurrence of various processes in the soil system, as Hedley et al [22] suggested, with the inorganic form of soil phosphorus, which is immobilized by soil microorganisms, contributing to the slower buildup of residual phosphorus, as approximately one quarter of bacterial-cell phosphorus from the soils is unextractable. It was concluded by Meason et al [49] that applied phosphorus fertilizers adsorbed onto the primary minerals can play an important role in increasing the HCl-Pi pool. In NX, the residual P concentrations were higher in the NPKM treatment compared to those at NC and JX, possibly due to excessive fertilization effects, which resulted in the saturation of P in the form of residuals that may be later converted to the moderately labile or labile phosphorus pools over time.

## Conclusions

The long-term addition of manure with inorganic fertilizer significantly increased the grain yield and labile P pool by improving the soil organic matter content and decreased the nonlabile P pool compared with the NPK and CK treatments. This study demonstrated that SOM is the key soil factor for improving P fertility and the combined application of manure with chemical fertilizer is not only a better strategy for managing P in the paddy soil but also has a positive impact on the physicochemical properties of the soil. In JX, the NPKM increased the apparent P balance more than NPK, due to a high fertilizer application rate, but in NC and NX, the apparent P balance in the NPK was higher than NPKM; therefore, the rate of combined application of chemical fertilizer and manure application should be lower than the rate of chemical fertilizer inputs to prevent the waste of resources and environmental P losses.
